# High sucrose consumption decouples intrinsic and synaptic excitability of AgRP neurons without altering body weight

**DOI:** 10.1038/s41366-023-01265-w

**Published:** 2023-02-01

**Authors:** Austin C. Korgan, Klausen Oliveira-Abreu, Wei Wei, Sophie L. A. Martin, Zoey J. D. Bridges, José Henrique Leal-Cardoso, Catherine C. Kaczorowski, Kristen M. S. O’Connell

**Affiliations:** 1 The Jackson Laboratory for Mammalian Genetics, Bar Harbor, ME USA; 2 Graduate School of Biomedical Science and Engineering, University of Maine, Orono, ME USA; 3 Neuroscience Program, Graduate School of Biomedical Science, Tufts University School of Medicine, Boston, MA USA; 4 Present Address: Instituto Superior de Ciências Biomédicas, Universidade Estadual do Ceará, Fortaleza, CE Brazil; 5 Present Address: Georgia State University, Atlanta, GA USA

**Keywords:** Hypothalamus, Homeostasis

## Abstract

**Background/Objective:**

As the obesity epidemic continues, the understanding of macronutrient influence on central nervous system function is critical for understanding diet-induced obesity and potential therapeutics, particularly in light of the increased sugar content in processed foods. Previous research showed mixed effects of sucrose feeding on body weight gain but has yet to reveal insight into the impact of sucrose on hypothalamic functioning. Here, we explore the impact of liquid sucrose feeding for 12 weeks on body weight, body composition, caloric intake, and hypothalamic AgRP neuronal function and synaptic plasticity.

**Methods:**

Patch-clamp electrophysiology of hypothalamic AgRP neurons, metabolic phenotyping and food intake were performed on C57BL/6J mice.

**Results:**

While mice given sugar-sweetened water do not gain significant weight, they do show subtle differences in body composition and caloric intake. When given sugar-sweetened water, mice show similar alterations to AgRP neuronal excitability as in high-fat diet obese models. Increased sugar consumption also primes mice for increased caloric intake and weight gain when given access to a HFD.

**Conclusions:**

Our results show that elevated sucrose consumption increased activity of AgRP neurons and altered synaptic excitability. This may contribute to obesity in mice and humans with access to more palatable (HFD) diets.

## Introduction

While significant public health efforts have been made to combat the growing prevalence of obesity, recent evidence shows that these efforts have failed to slow this progression [1]. Recent studies have identified central nervous system (CNS) pathways as a key driver contributing to increased caloric intake and body weight gain [2–6]. The American Medical Association has recognized obesity as a disease since 2013 [7], but few therapeutic treatments have succeeded in reducing body weight or diet-induced obesity (DIO) in human patients [8]. The most efficacious treatments target the hypothalamus and melanocortin system, specifically glucagon-like protein 1 receptor (GLP-1R) and melanocortin 4 receptor (MC4R) agonists [9–11]. While these new pharmacological treatments are encouraging, diet and exercise remain the safest and most common lifestyle interventions; however, results are often temporary and many individuals regain weight in less than 5 years [12–18]. Understanding obesogenic factors that alter CNS function may allow for development of interventions that ‘reset’ these mechanisms and promote sustained weight loss through reduced appetite.

The role of AgRP/NPY neurons as signal integrators of peripheral and central cues to drive feeding and behavior has been studied extensively in the context of normal chow fed (NCD) [19–23], caloric restriction (fasting) [24–26], and high-fat diet (HFD) [24, 27–31]. Further, this hypothalamic neuron population interacts with midbrain dopamine neurons to modulate response to rewarding stimuli (e.g. palatable food and drugs) [32]. Recent studies have identified AgRP neuronal activation and synaptic plasticity responsible for driving feeding in response to fasting [26, 31]. Similarly, we and others have recently described acute and long-term synaptic mechanisms [31] that mediate AgRP neuronal hyperactivity ex vivo [24, 28] and postprandial desensitization identified by in vivo calcium imaging studies [29, 30]. While recent studies have identified distinct gut-brain pathways for fat and sugar signaling to AgRP/NPY neurons [33–35] the long-term influence of high dietary sucrose consumption on AgRP neuronal function, plasticity, and body weight has not been explored.

Recent research has identified functional behavioral and metabolic differences between HFD, high sucrose diet, and liquid sucrose (SucrW) consumption [36, 37]. Generally, long-term SucrW consumption does not result in significant changes in body weight, caloric intake, or glucose and insulin processing [37–39]. Separate circuitries regulate the homeostatic and hedonistic rewards associated with sugar consumption [40]. Regulation of Sucrose consumption by peripheral hormones, including ghrelin, leptin, and insulin, suggest that CNS and AgRP neuronal signal integration mechanisms coordinate homeostatic consumption of Sucrose diet [38, 41–43]. Further, ‘motivated’ (non-homeostatic or hedonistic) SucrW consumption demonstrates ‘top-down’ processing within the CNS and is associated with disrupted reward processing and diminished valence of the stimulus reward [44] likely linked to plasticity within the hypothalamus [45, 46] but could also be regulated by taste receptors [47–49] or leptin and insulin signaling [43, 50]. Further, control of Sucrose intake by AgRP (and POMC) neuronal output has been shown within the melanocortin system, where α-MSH and AgRP have opposite effects on SucrW consumption [51–53]. However, specific changes in AgRP neuronal intrinsic excitability and synaptic plasticity following long-term SucrW consumption have not been described.

In the current study, we investigated the impact of long-term SucrW consumption on AgRP neuronal function and adaptation in the absence of body weight gain. We identified a SucrW-dependent increase in intrinsic excitability, though not as robust as that seen in HFD fed mice, along with an increase in inhibitory post synaptic currents (mIPSC), replicating a decoupling between AgRP neuronal activity and GABAergic synaptic inputs previously identified in DIO mice [31]. Additionally, we found that leptin-mediated inhibition of AgRP neurons was attenuated independent of weight gain, along with a SucrW priming effect for DIO that further amplified AgRP neuronal activity. Together, these findings highlight a mechanism through which high Sucrose consumption primes an individual for increased caloric intake by remodeling AgRP neurons comparable to DIO. While dietary macronutrients (i.e. fat and sugar) engage divergent pathways to communicate with the CNS, we show that alterations to hypothalamic circuitry, specifically AgRP neurons, follow similar mechanistic responses and may present attractive treatment options, especially in an obesogenic food environment that promotes over consumption of many macronutrients [5, 54–56].

## Methods

### Animals

**Fig. 1:**
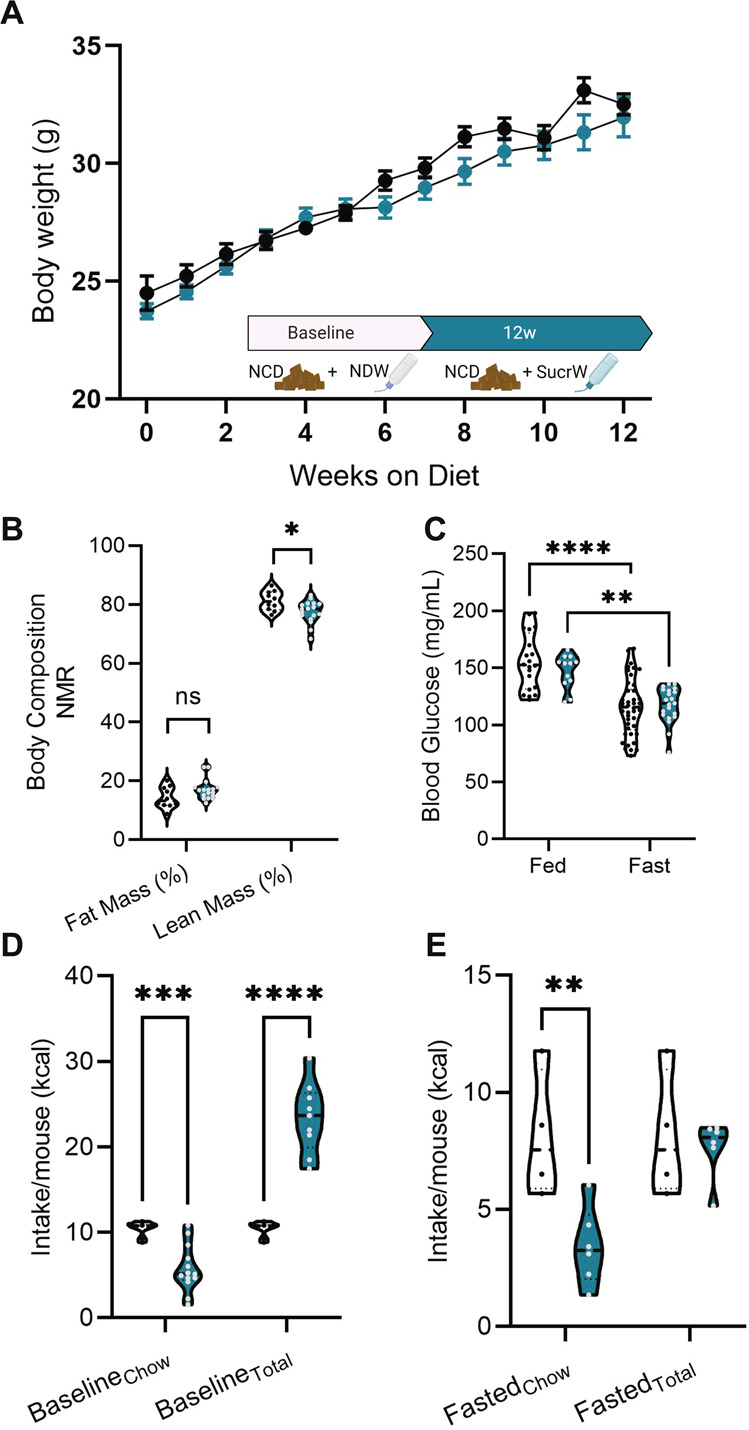
Male mice are resistant to changes in bodyweight gain, body composition, and locomotor activity despite increased caloric intake on high Sucrose diet. **A** Cumulative body weight curves for male mice during 12 weeks of NDW (*n* = 22) or SucrW (*n* = 57) feeding and feeding timeline (inset) **B** Body fat (%) was not significantly different between NDW (*n* = 10) and SucrW (*n* = 17) but lean mass (%) was slightly reduced in SucrW (*n* = 17) mice compared to NDW (*n* = 10) mice. **C** Blood glucose (mg/mL) from fed and fasted NDW (fed: *n* = 20, fast: 42) and SucrW mice (fed: *n* = 12, fast: 21). **D** Baseline chow (NDW: *n* = 8, SucrW: 13) and total intake (NDW: *n* = 8 SucrW: 9) (kcal) per pen/mouse during a 24 h period following 10 weeks of NDW or SucrW feeding. **E** Chow (NDW: *n* = 4, SucrW: 6) and total intake (NDW: *n* = 4, SucrW: 6) (kcal) per pen/mouse during a 6 h period, following a 12 h fast. For all violin plots, dashed line indicates median, dotted lines indicate quartiles. For all analysis, diet conditions compared to NDW using standard *t*-test or parametric ANOVA with a *post hoc* Tukey’s multiple comparisons test and a mixed-effects RM ANOVA for repeated measures (*p < 0.05; **p < 0.01; ***p < 0.001).

The transgenic strain *hrGFP-NPY* (JAX Stock #006417) and C57Bl/6 J (JAX Stock #000664) were used in this study. Founder mice were obtained from the JAX Repository and maintained by backcrossing with C57Bl/6 J. hrGFP-NPY mice were SNPtyped to confirm that the strain is on a congenic C57Bl/6 J background except for the transgene insertion site on Chr7. All animal care and experimental procedures were approved by The Animal Care and Use Committee at The University of Tennessee Health Science Center and The Jackson Laboratory. Mice were maintained at 22–24 °C on a 12 h:12 h light/dark cycle (lights on at 0600–1800). All mice used for breeding were fed standard lab chow (UTHSC–Teklad 7912: 3.1 kcal/g metabolizable energy, 17 kcal% fat or JAX–LabDiets 5K0Q: 3.15 kcal/g metabolizable energy, 16.8 kcal% from fat). There was no significant effect of control diet manufacturer (Teklad v LabDiets) or performance site (UTHSC v JAX), so data from both sites were combined as described previously [31]. Mice were weaned at 21 days and group housed with same-sex litter mates (n = 2–5 mice/pen). At 8 weeks of age, experimental mice were randomly assigned to either normal drinking water (NDW) or to a high-Sucrose group (SucrW; Sucrose (Fisher Scientific; S250)) in which the standard acidified water was replaced with water sweetened with 10% (w/v) Sucrose (0.52 kcal/g metabolizable energy) [39, 43] for 10–12 weeks; no other water was available to the SucrW group and all mice were fed a NCD (Fig. 1A, inset). The same Sucrose concentration was used at both UTHSC and JAX and there was no significant effect of site on response to Sucrose water, so data from both sites were combined.

Separate cohorts of mice were given access to SucrW for only 2 days and were then fed a high-fat diet (HFD; Research Diets D12451: 4.73 kcal/g metabolizable energy, 45 kcal% from fat); HFD available *ad libitum* for mice randomly assigned to the 2-day (HFD_2d_; Fig. 6A) condition as previously described [28]. All water was available *ad libitum*. Mice were weighed weekly and used for experiments at 16–20 weeks of age.

### Open field test

Locomotor activity was measured in the open-field test (OFT). The OFT consists of solid white polyurethane foam panels (30.5 cm high) and a floor (40 cm × 40 cm square) illuminated at 150 lux. Locomotor activity was tracked by an overhead camera and analyzed with ANY-maze (Stoelting Co., version 7.14). Following >1 h habituation to the test room, mice were placed into the center square (10 cm × 10 cm) of the arena for a 10 min trial. Behaviors scored were distance travelled (m), thigmotaxis entries, thigmotaxis exits, total thigmotaxis time, center entries, center exits, and center time. Following each trial, the arena was cleaned with 70% ethanol.

### Feeding behavior

Feeding behavior was assessed by measuring food intake over four days. During the first three days, baseline NCD and NDW or SucrW consumption were collected by daily weighing. On the third day, NCD and SucrW were removed at 17:00 h. The next day, NCD and SucrW were returned at 0900 and intake was measured for 6 h. Body weights were measured pre- and post-fast and post-refeeding. Food intake is represented as total kcal consumed via NCD, HFD, and SucrW per pen divided by the total number of mice per pen. All mice were group housed to avoid isolation-induced stress and potential changes in feeding behavior [57–59].

### Body composition, blood glucose, and liver weight

Body composition of mice was measured at baseline (8 weeks old) and following 10–12 weeks of NDW or SucrW feeding (18–20 weeks old). All mice were *ad lib* fed at the time of body composition measurements. Mice were placed in a plastic cylinder (50 mm diameter) and inserted into the nuclear magnetic resonance (NMR) instrument (Bruker Minispec LF50, Billerica, MA USA) for the duration of the scan (<2 min).

Blood glucose was measured following 9–11 weeks of NDW or SucrW feeding (17–19 weeks old). Mice were tested in both fed and fasted groups with random order of testing for both timepoints. For all groups, blood was collected between 8–10 AM. For blood glucose measurements, a small nick in the tail was made with scissors and a drop of blood was collected on a clean blood glucose test strip and inserted into the blood glucose meter (TRUEtest, Trividia Health, FL USA).

Whole livers were collected and weighed during the harvest for brain slice electrophysiology experiments as described below.

### Electrophysiology

#### Slice preparation

For all experiments, brain slices were prepared between 0900 and 1030. Mice (16–17 weeks old) used for electrophysiology experiments were deeply anesthetized using isoflurane before decapitation and rapid removal of the brain. The brain was then submerged in ice-cold, oxygenated (95% O_2_/5% CO_2_) cutting solution (in mM: 119 NaCl, 90 Sucrose, 2.5 KCl, 1 MgSO_4_, 2 CaCl_2_, 1.25 NaH_2_PO_4_, 23 NaHCO_3_, and 10 glucose). Coronal slices (250 μm) were cut using a vibratome (VT1000S, Leica) and incubated in oxygenated aCSF (in mM: 119 NaCl, 2.5 KCl, 1 MgSO_4_, 2 CaCl_2_, 1.25 NaH_2_PO_4_, 23 NaHCO_3_, and 10 glucose) for at least 1 h prior to recording.

#### Slice recording

Slices were transferred to a recording chamber constantly perfused (~2 ml/min) with oxygenated aCSF. GFP-positive AgRP/NPY neurons were identified using epifluorescence and standard GFP filters on a fixed-stage Scientifica (Uckfield, UK) SliceScope 1000 microscope equipped with a digital camera (Q-Imaging, Surry, BC, Canada). All recordings were performed using a Multiclamp 700B amplifier and Digidata 1550 A, controlled using Clampex 10.7 (Molecular Devices, San Jose, CA, USA). Data were digitized at 20 kHz and filtered at 5 kHz using the built-in four-pole Bessel filter of the Multiclamp 700B.

Recording pipettes were pulled from filamented thin-wall borosilicate glass (TW150F-4, World Precision Instruments) and had a resistance of 4-7 MΩ when filled with internal solution (for intrinsic excitability (AP) recordings, in mM: 130 K-gluc, 10 KCl, 0.3 CaCl_2_, 1 MgCl_2_, 1 EGTA, 3 MgATP, 0.3 NaGTP, and 10 HEPES, pH 7.35 with KOH; for synaptic recordings, in mM: 140 KCl, 0.3 CaCl_2_, 1 MgCl_2_, 1 EGTA, 3 MgATP, 0.3 NaGTP, and 10 HEPES, pH 7.35 with KOH). The liquid junction potential (LJP) between normal aCSF and the K-gluconate solution used for intrinsic recordings was +14.7 mV and was corrected. The LJP between aCSF and the KCl intracellular solution was +4.75 mV and was not corrected.

Whole-cell current clamp recordings of resting membrane potential and spontaneous firing were recorded in the presence of DNQX (10 μM; Tocris) and picrotoxin (100 μM; Tocris). For experiments testing inhibition of AgRP neurons by leptin, mice were fasted overnight to promote increased intrinsic excitability and 100 nM leptin (Tocris; 116–130) was bath applied. Whole-cell voltage-clamp recordings of mEPSC and mIPSC were conducted in the presence of TTX (1 µM; Tocris) and Picrotoxin (100 uM; Tocris) for mEPSCs and DNQX (10 µM; Tocris) for mIPSCs.

#### Data analysis and statistics

Post-synaptic current frequencies, amplitudes, inter-event intervals and τ decay were measured using Axograph (AxoGraph, Inc). Statistical outliers were identified using the ROUT method (Q = 1% cutoff threshold) as implemented in GraphPad Prism 9. Group differences were analyzed with two-way ANOVA followed by Tukey’s multiple comparisons *post hoc* test or unpaired t-tests using Prism 9 (GraphPad). Variance between groups was measured with either an F (*t*-tests) or Bartlett’s (ANOVA) test. Contingency tables were analyzed for group differences with Fisher’s exact test. For cumulative distribution of mEPSC and mIPSC amplitudes, group differences were compared with the nonparametric Kolgoromov–Smirnoff test. For repeated measures analysis, group differences were analyzed by two-way RM-ANOVA using Prism 9 (GraphPad). Data visualization was performed using Prism or r/ggplot2 (version 4.2.1). For all statistical tests, a value of *p* < 0.05 was considered significant. All analyses were conducted by an experimenter blinded to treatment group. Data are presented as the mean ± SEM; violin plots are presented as median ± quartile. For all experiments, a priori estimation of sample sizes was performed using statistical tools in G*Power3.1. The partial η^2^ from previous experiments was used to estimate effect sizes for α = 0.05 and power (1-β) = 0.95. For detailed ANOVA and t-test results, see Table 1.

**Table 1:** Full statistical details for ANOVA and *t*-test results.

Figure	*n*	*n* unit	F_(DFn, DFd)_ or t_(df)_	*p*
1A	NDW = 22, SucrW = 57	mice	0.7845_(12,744)_	0.6669
S1A	NDW = 9, SucrW = 39	mice	0.541_(46)_	0.591
S1B	NDW = 6, SucrW = 33	mice	1.082_(37)_	0.286
1B	NDW = 10, SucrW = 17	mice	Fat = 1.974_(25)_; Lean = 2.580_(25)_	0.0596 0.0162
1C	NDW_fed_ = 14, SucrW_fed_ = 12, NDW_fast_ = 31, SucrW_fast_ = 31	mice	Diet = 0.4081_(1,91)_ Fast = 48.74_(1,91)_	0.5245 <0.0001
S1C	NDW_fed_ = 9, SucrW_fed_ = 6, NDW_fast_ = 9, SucrW_fast_ = 4	mice	9.86_(1,24)_	0.0044
S1D	NDW_fed_ = 3; SucrW_12Wk_ = 3; HFD_8Wk_ = 4		4.555_(2,9)_	<0.0001
S1E	NDW = 7, SucrW = 9	mice	0.0296_(14)_	0.9768
S1F	NDW = 7, SucrW = 9	mice	1.71 _(14)_	0.1094
1D Chow	NDW = 8, SucrW = 13	pens	4.662_(19)_	0.0007
1D Total	NDW = 8, SucrW = 9	pens	7.523_(13)_	<0.0001
1E Chow	NDW: *n* = 4; SucrW: *n* = 6	pens	3.471_(8)_	0.0046
1E Total	NDW: *n* = 4; SucrW: *n* = 6	pens	0.3965_(8)_	0.9156
S2A	NDW: *n* = 10; SucrW: *n* = 10	mice	1.207_(12,216)_	0.2798
S2B	NDW: *n* = 6; SucrW: *n* = 6	pens	4.439_(5)_	0.0013
S2C	NDW: *n* = 6; SucrW: *n* = 6	pens	8.194_(5)_	<0.0001
S2D	NDW: *n* = 13; SucrW: *n* = 14	cells	0.5412_(12)_	0.5932
S2E	NDW: *n* = 13; SucrW: *n* = 14	cells	1.202_(12)_	0.2407
2B	NDW_fed_ = 44, NDW_fast_ = 21, SucrW_12Wk_ = 62, SucrW_12Wk+Fast_ = 11	cells	Diet = 3.272_(1,134)_ Fast = 6.973_(1,134)_	0.0727 0.0093
2C	NDW_fed_ = 44, NDW_fast_ = 21, SucrW_12Wk_ = 62, SucrW_12Wk+Fast_ = 11	cells	Diet = 0.2979 _(1,134)_ Fast = 0.6521_(1,134)_	0.5861 0.4208
S3B	NDW_fed_ = 21, NDW_fast_ = 19, SucrW_12Wk_ = 53, SucrW_12Wk+Fast_ = 11	cells	Diet = 1.138_(1,101)_ Fast = 1.674_(1,101)_	0.2886 0.1986
S3D	NDW_fed_ = 38, NDW_fast_ = 15, SucrW_12Wk_ = 44, SucrW_12Wk+Fast_ = 7	cells	Diet = 3.272_(1,134)_ Fast = 6.973_(1,134)_	0.0727 0.0093
3B	NDW_fast_ = 9, SucrW_12Wk_ = 6	cells	NDW = 4.6677_(8)_ SucrW = 1.942_(5)_	0.0016 0.1098
3C	NDW_fast_ = 9, SucrW_12Wk_ = 6	cells	NDW = 3.724_(8)_ SucrW = 1.367_(5)_	0.0029 0.1149
3D	NDW_fed_ = 5; SucrW_12Wk_ = 3; HFD_8Wk_ = 4	mice	37.87_(2,9)_	<0.0001
4B	NDW = 9, SucrW_12Wk_ = 9	cells	0.169_(16)_	0.0868
S4A	SucrW_12Wk_ Low *f*_mEPSC(s-1)_ = 5, SucrW_12Wk_ High *f*_mEPSC(s-1)_ = 4	cells	10.84_(7)_	<0.0001
4C	NDW = 9, SucrW_12Wk_ = 9	cells	2.156_(16)_	0.048
4D	NDW = 9, SucrW_12Wk_ = 9	cells	1.21_(16)_	0.244
4E	NDW = 9, SucrW_12Wk_ = 9	cells	2.561_(16)_	0.021
4F	NDW = 9, SucrW_12Wk_ = 9	cells		
4G	NDW = 9, SucrW_12Wk_ = 9	cells		
5B	NDW = 6, SucrW_12Wk_ = 8	cells	2.854_(12)_	0.015
5C	NDW = 6, SucrW_12Wk_ = 8	cells	2.857_(12)_	0.014
5D	NDW = 6, SucrW_12Wk_ = 8	cells	0.024_(12)_	0.981
5E	NDW = 6, SucrW_12Wk_ = 8	cells	1.531_(12)_	0.152
5F	NDW = 6, SucrW_12Wk_ = 8	cells		
5G	NDW = 6, SucrW_12Wk_ = 8	cells		
6B	NDW = 6, SucrW = 6	mice	6.092_(10)_	0.0001
6C	NDW = 6, SucrW = 6	mice	6.909_(10)_	<0.0001
6D	NDW = 10, SucrW_2d+2d HFD_ = 8, SucrW_12Wk+2d HFD_ = 4, HFD_2d_ = 7	mice	20.65_(3,25)_	<0.0001
6E	NDW = 9, SucrW_2d_ = 10, SucrW_2d+2d HFD_ = 12, SucrW_12Wk+2d HFD_ = 11	mice	8.287_(3,40)_	0.0002
6F	NDW = 9, SucrW_2d_ = 5, SucrW_2d+2d HFD_ = 7, SucrW_12Wk+2d HFD_ = 4	mice	8.510_(3,21)_	0.0007
S5A	NDW = 4, SucrW_2d_ = 4, SucrW_2d+2d HFD_ = 7, SucrW_12Wk+2d HFD_ = 4	mice	2.466_(3,15)_	0.3323
S5B	NDW = 9, SucrW_2d+2d HFD_ = 11, SucrW_12Wk+2d HFD_ = 10	mice	3.663_(2,27)_	0.0391
6G	NDW_fed_ = 17, SucrW_2d_ = 24, SucrW_2d+2d HFD_ = 16, SucrW_12Wk+2d HFD_ = 13	cells	3.111_(3,66)_	0.0322
6H	NDW_fed_ = 17, SucrW_2d_ = 24, SucrW_2d+2d HFD_ = 16, SucrW_12Wk+2d HFD_ = 13	cells	0.1740_(3, 66)_	0.9136
6K	SucrW_2d+2d HFD_ = 8	cells	3.656_(7)_	0.0081
6M	SucrW_12Wk+2d HFD_ = 8	cells	1.027_(7)_	0.3388

*n* number of mice or cells (designated by *n* unit), F_(DFn, DFd)_ = ANOVA *F* value and Degrees of Freedom for the numerator or denominator, respectively, and *p* = *p* value for a given statistic.

## Results

### Male mice are resistant to changes in bodyweight gain, body composition, and locomotor activity despite increased caloric intake on high sucrose diet

To quantify the impact of high dietary sucrose on weight gain and obesity, mice were weighed weekly throughout the Sucrose diet feeding. Consistent with previous reports [37–39], there was no difference in body weight between NDW and SucrW mice (Fig. 1A) at any point following sucrose administration (Fig. S1A, B). Female mice were also resistant to weight gain following SucrW consumption (Fig. S2A) and based on previous studies, we predict dissimilar metabolic and synaptic mechanisms that will require future investigation [31, 60–63]. NMR for body composition revealed no difference in fat mass in SucrW mice compared to NDW controls but did have a decrease in lean mass (Fig. 1B). There was a main effect of fasting on blood glucose, which was decreased in both SucrW and NDW fasted mice (Fig. 1C). Liver weight was elevated in SucrW_fed_ mice compared to NDW_fed_, NDW_fast_, and SucrW_fast_ mice (Fig. S1C). The OFT did not reveal differences in locomotor or anxiety-like behavior between NDW and SucrW mice (Fig. S1D, E). Baseline caloric intake from chow was decreased in SucrW_fed_ mice compared to NDW_fed_, however total caloric intake (chow + SucrW water) was increased in SucrW_fed_ mice (Fig. 1D). Upon refeeding following a 16 h fast (with access to NDW during the fast), SucrW_fast_ mice consumed less chow than NDW_fast_ mice but equivalent total calories (Fig. 1E).

### Long-term high sucrose intake increases intrinsic activity in AgRP neurons and decreases leptin sensitivity

**Fig. 2:**
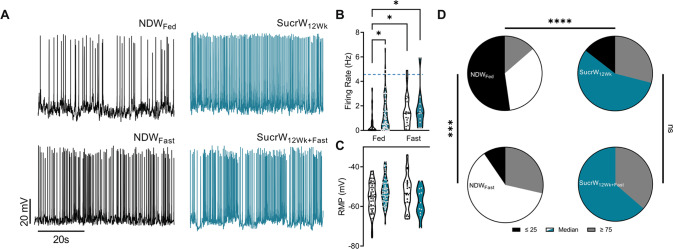
Long-term high Sucrose diet increases intrinsic activity in AgRP neurons and decreases leptin sensitivity in male mice. **A** Representative traces of fed and fasted NDW and SucrW mice **B** AgRP neuronal firing rate was increased in SucrW_12Wk_ (*n* = 62) and NDW_Fast_ (*n* = 21) and SucrW_12Wk+Fast_ (*n* = 11) mice compared to NDW_Fed_ (*n* = 44). **C** There was no effect of diet or fast on RMP. **D** Contingency tables from fed and fasted NDW and SucrW mice. NDW_Fed_ mice had an altered proportion of silent (F_AP_ < 25 percentile; 0.08659 Hz), medium firing (F_AP_ > 25 and < 75 percentile), and high firing (F_AP_ > 75 percentile; 1.75 Hz) cells compared to NDW_Fast_ and SucrW_12Wk_ and there was no difference between SucrW_12Wk_ and SucrW_12Wk+Fast_. NDW_fed_: F_AP_ < 25 percentile = 52.27% (*n* = 23), F_AP_ > 25 percentile and <75 percentile = 34.09% (n = 15), and F_AP_ > 75 percentile = 13.64% (*n* = 6); NDW_fast_: F_AP_ < 25 percentile = 9.52% (*n* = 2), F_AP_ > 25 percentile and <75 percentile = 61.9% (*n* = 13), and F_AP_ > 75 percentile = 28.57% (*n* = 6), *p* = 0.0004; SucrW_12Wk_: F_AP_ < 25 percentile = 14.52% (*n* = 9), F_AP_ > 25 percentile and <75 percentile = 56.45% (*n* = 35), and F_AP_ > 75 percentile = 29.03% (*n* = 18), *p* < 0.0001; SucrW_12Wk+Fast_: F_AP_ < 25 percentile = 0% (*n* = 0), F_AP_ > 25 percentile and <75 percentile = 63.64% (*n* = 7), and F_AP_ > 75 percentile = 36.36% (*n* = 4), (*p* = 0.3864). For all violin plots, dashed line indicates median, dotted lines indicate quartiles. For all analysis, diet conditions compared to NDW using standard parametric ANOVA with a *post hoc* Tukey’s multiple comparisons test or Fisher’s Exact test (*p < 0.05; **p < 0.01; ***p < 0.001).

Commonly utilized rodent HFD (Research Diets, D12451) contains both added sucrose (17 kcal%) and increased fat content, even when compared to other commonly used diets such as ResearchDiets D12492 (7 kcal% sucrose). We previously demonstrated that a high-fat/high-sugar diet rapidly and persistently induced an increase in intrinsic excitability of AgRP neurons [24, 28–31], so we were interested in determining the extent to which elevated dietary sucrose alone impacts AgRP neuronal function. As previously reported [24, 26, 28, 31, 64], in *ad libitum* fed control mice (NCD + NDW), the average baseline firing rate of AgRP neurons is low (<1 Hz, Fig. 2B) and is significantly increased following an overnight fast (Fig. 2B). There were significant main effects of diet and fasting; both 16 h fast and SucrW_12Wk_ consumption resulted in increased firing rates in AgRP neurons (Fig. 2A, B). There was no interaction between fasting and sucrose, as the mean firing rate was not significantly different between the NDW and SucrW_12Wk_ groups after an overnight fast. There was no difference in resting membrane potential (RMP) (Fig. 2C).

A significant difference in the firing rate of AgRP neurons following long-term sucrose consumption may arise due to either (1) an overall increase in neuronal output across the population due an increase in the maximal rate or (2) a shift in the distribution of rates to higher frequencies within the existing bounds of the underlying distribution. To determine which potential mechanism underlies the significant difference in mean firing rates with SucrW_12Wk_, we evaluated the distribution of firing rates within each group. Fisher’s exact test identified a significant decrease in the proportion of silent (F_AP_ < 25 percentile; 0.08659 Hz), medium firing (F_AP_ > 25 and <75 percentile), and high firing (F_AP_ > 75 percentile; 1.75 Hz) AgRP neurons from NDW_fast_ (Fig. 2D; *p* = 0.0004) and SucrW_12Wk_ (Fig. 2D; *p* < 0.0001) mice compared to NDW_fed_ mice and no difference between SucrW_12Wk_ and SucrW_12Wk+Fast_ mice (Fig. 2D; *p* = 0.3864). Frequency distribution plots of the firing rates of all cells (Fig. S3A) suggest that the *maximal* firing rate of AgRP neurons is not altered by sucrose, rather that sucrose induces a shift in the center of the distribution to higher values without changes the maximal firing rate of AgRP neurons. Consistent with this, when we stratify the entire dataset into quartiles and remove the bottom 25% of firing rates (F_AP_ < 0.08659 Hz), there is no longer a significant main effect of either Sucrose or fasting. However, if we repeat this same analysis but instead remove the top quartile of values (F_AP_ > 1.75 Hz), the significant main effects of both Sucrose and Fasting remain, suggesting that neither chronic sucrose consumption nor fasting impact the *maximal* firing rate of AgRP neurons, rather each of these interventions causes a general shift of AgRP neuronal firing to higher values within the bounds of the original distribution as established from NDW mice. While the activity of AgRP neurons from DIO mice is largely uniform, with the entire population shifting to a higher rate of activity [24], our results indicate that the response to SucrW is more variable, with only a subset of neurons exhibiting hyperexcitability, suggesting that the response of AgRP neurons to diet is sensitive to macronutrient content.

**Fig. 3:**
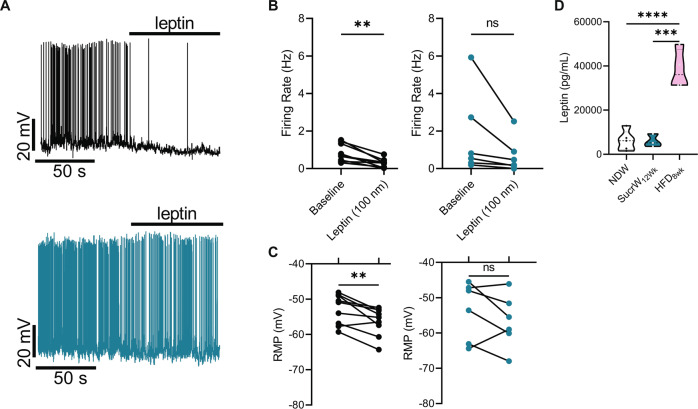
Long-term Sucrose consumption attenuates leptin inhibition of AgRP neuronal activity in male mice. **A** Representative traces of NDW_Fast_ (top) or SucrW_12Wk_ (bottom) with bath Leptin (100 nm) application. **B** Firing rate of NDW_Fast_ (*n* = 9; left) and SucrW_12Wk_ (*n* = 6; right) before and after bath leptin application. **C** Leptin application resulted in a hyperpolarization of the RMP in NDW_fast_ mice, no change was seen in SucrW_12Wk_ mice after bath leptin application. **D** Plasma leptin levels (pg/mL) were not changed in SucrW_12Wk_ mice (*n* = 3) compared to NDW mice (*n* = 5) and unlike HFD_8Wk_ mice (*n* = 4). For all violin plots, dashed line indicates median, dotted lines indicate quartiles. Statistical comparisons using *t*-tests (*p < 0.05; **p < 0.01; ***p < 0.001, ****p < 0.0001).

Leptin inhibition of AgRP neuronal firing has been well defined [65], and leptin resistance likely contributes to the DIO-associated hyperexcitability of AgRP neurons in obese mice [24]. As expected, 100 nm leptin inhibited AgRP neurons from NDW_fast_ mice (Fig. 3A, B), due in part to a significant hyperpolarization of the resting membrane potential (Fig. 3A, C). However, we did not observe a significant effect of leptin on either neuronal firing or membrane potential in SucrW_12Wk_ mice, similar to effects identified in DIO mice [24] (Fig. 3A, C). However, the unidirectionality of the response of AgRP neurons to leptin suggests that SucrW feeding results in an attenuated leptin response rather than overt leptin resistance. Further, unlike HFD mice, SucrW_12Wk_ mice did not have elevated plasma leptin (Fig. 3D) or insulin (S1F) compared to NDW_fed_ mice, consistent with SucrW_12Wk_ mice having fat mass comparable to lean controls (Fig. 1B).

### Long-term sucrose consumption does not alter excitatory synaptic input to AgRP neurons

**Fig. 4:**
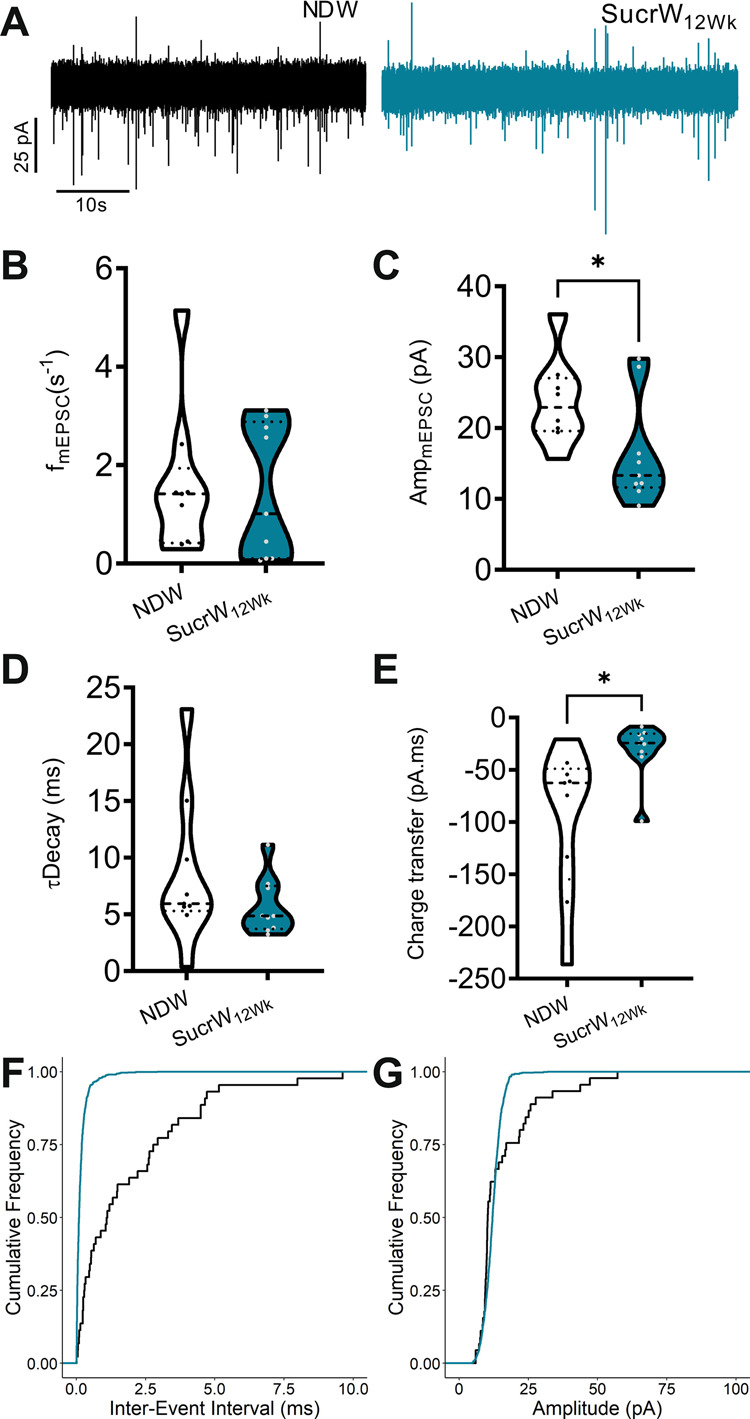
Long-term Sucrose consumption does not alter excitatory synaptic input to AgRP neurons in male mice. **A** Representative traces of mEPSCs onto AgRP neurons in fed or fasted male mice on either NDW or SucrW_12Wk_. **B** Mean mEPSC frequency onto AgRP neurons from NDW (*n* = 9) or SucrW_12Wk_ (*n* = 9). **C** Mean mEPSC amplitude in AgRP neurons. **D** Mean tDecay in AgRP neurons from NDW or SucrW_12wk_ mice. **E** Mean charge transfer in AgRP neurons from NDW or SucrW_12Wk_ mice. **F** Cumulative frequency of inter-stimulus interval intervals in AgRP neurons from NDW or SucrW_12Wk_ mice. **G** Cumulative frequency of amplitudes in AgRP neurons from NDW or SucrW_12Wk_ mice. For all violin plots, dashed line indicates median, dotted lines indicate quartiles. Statistical comparisons using *t*-tests and cumulative frequencies compared using Kolgoromov-Smirnoff test (*p < 0.05).

Given the persistent hyperexcitability of AgRP neurons following SucrW_12Wk_ consumption, we next examined whether these diet-induced changes in firing rate were associated with increased excitatory neurotransmission, as seen in NDW_fast_ mice [31, 66, 67]. When we investigated the impact of long-term SucrW consumption on the frequency of excitatory inputs to AgRP neurons (*f*_mEPSC_), we found no difference in the *f*_mEPSC_ (Fig. 4A, B), similar to what we previously reported in DIO mice [31]. However, the *f*_mEPSC_ from SucrW mice was not normally distributed (Shapiro–Wilk test; W = 0.806, *p* = 0.024), with a significant difference between the low and high *f*_mEPSC_ SucrW groups (Fig. S4A). Notably, there was representation of each mouse (*n* = 3) in both the low and high *f*_mEPSC_ groups (Fig. S4B), with some cells exhibiting a significant decrease in the *f*_mEPSC_, while excitatory input to others is unchanged, suggesting that a heterogeneous population of presynaptic inputs [68] is differentially responding to dietary sucrose.

When we investigated the impact of SucrW consumption on the amplitude of mEPSCs, we identified a small decrease in amplitude of mEPSC (Fig. 4C) with no change in decay constant (_T_decay) (Fig. 4D), resulting in a small increase in charge transfer (Fig. 4E). The left-shifted distribution of cumulative mEPSC amplitudes reflects the decrease in amplitude between groups (Fig. 4G). Together, these results suggest that AgRP neurons receive excitatory inputs from presynaptic neurons with differing responses to increased SucrW consumption and that SucrW consumption may influence postsynaptic response to excitatory input onto AgRP neurons.

### Long-term sucrose consumption alters inhibitory synaptic input to AgRP neurons

**Fig. 5:**
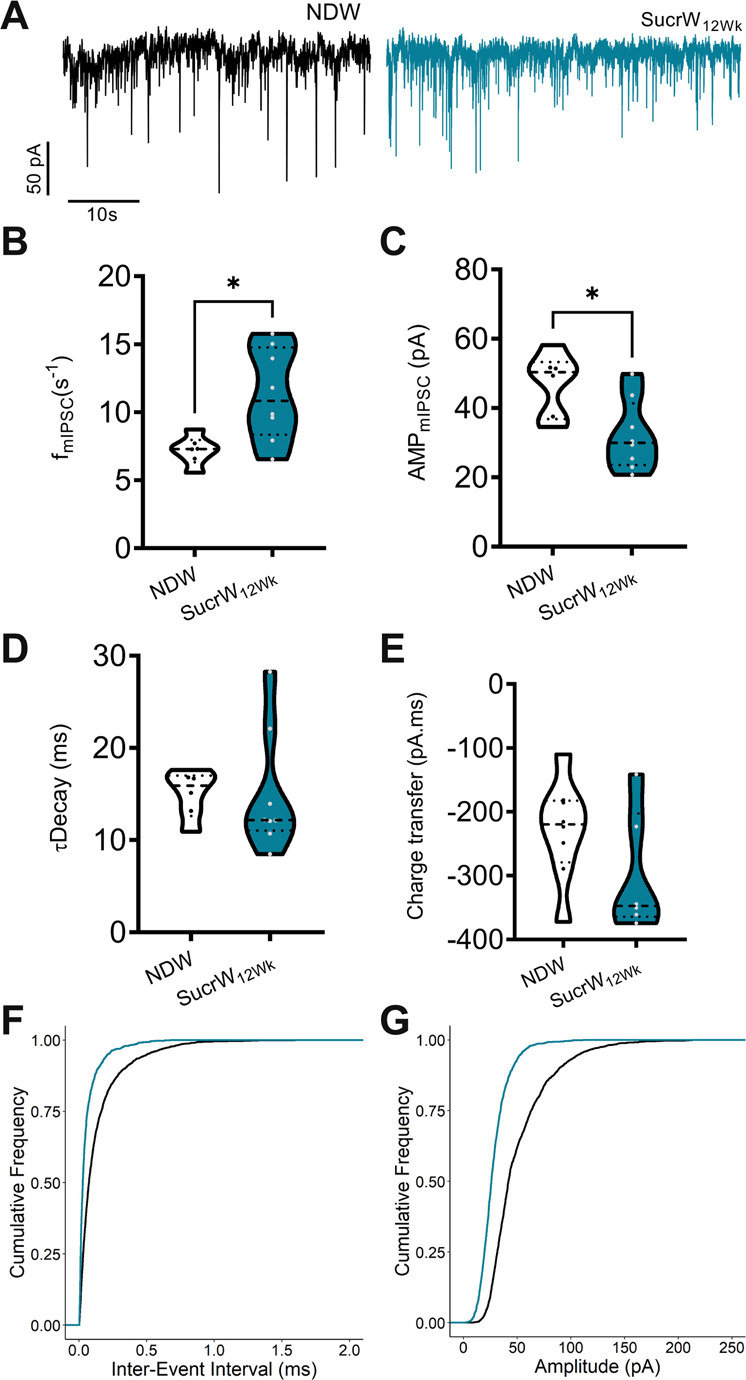
Long-term Sucrose consumption alters inhibitory synaptic input to AgRP neurons in male mice. **A** Representative traces of mIPSCs onto AgRP neurons in fed or fasted male mice on either NDW or SucrW_12Wk_. **B** Mean mIPSC frequency onto AgRP neurons from NDW (*n* = 6) or SucrW_12Wk_ (*n* = 8) males: *n* = 6–8 (neurons/group). **C** Mean mIPSC amplitude in AgRP neurons. **D** Mean tDecay in AgRP neurons from NDW or SucrW_12wk_ mice. **E** Mean charge transfer in AgRP neurons from NDW or SucrW_12Wk_ mice. **F** Cumulative frequency of inter-stimulus interval intervals in AgRP neurons from NDW or SucrW_12Wk_ mice. **G** Cumulative frequency of amplitudes in AgRP neurons from NDW or SucrW_12Wk_ mice. For all violin plots, dashed line indicates median, dotted lines indicate quartiles. Statistical comparisons using *t*-tests and cumulative frequencies compared using Kolgoromov–Smirnoff test (*p < 0.05).

We identified significant differences in the *f*_mIPSC_ (Fig. 5A, B) and amplitude of mIPSC (Fig. 5C) but no significant difference in decay constant (_T_decay) (Fig. 5D) or charge transfer (Fig. 5E). Left-shifted distribution of cumulative inter-event intervals (IEI) reflect the increased *f*_mIPSC_ of SucrW_12Wk_ mice (Fig. 5F), despite an increase in the overall excitability of AgRP neurons from these mice (Fig. 2). The left-shifted distribution of mIPSC amplitudes reflects the difference between groups (Fig. 5G). Overall, these results suggest that SucrW_12Wk_ fed mice have altered synaptic inputs and decoupling from intrinsic firing rates, at least partially driven by leptin and GABA resistance. However, differences in bodyweight did not occur in SucrW_12Wk_ mice, suggesting that Sucrose induced decoupling of inhibition (via leptin and GABA) may prime an organism for DIO if presented with more palatable (HFD) options.

### Acute sucrose consumption does not alter AgRP neuronal activity but does drive increased food intake, bodyweight gain, and AgRP neuronal activity with acute HFD feeding

**Fig. 6:**
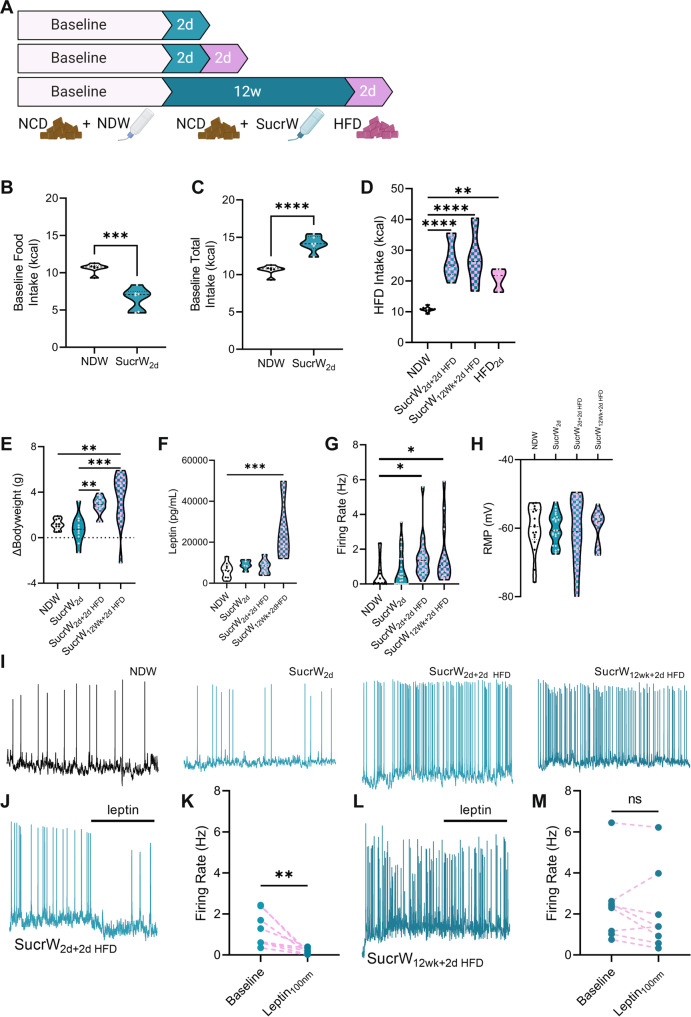
Acute Sucrose consumption does not alter AgRP neuronal activity but does drive increased food intake and AgRP neuronal activity with acute HFD feeding in male mice. **A** Timeline of feeding schedules for combined SucrW and acute HFD feeding experiments. **B** Baseline food (NCD) intake per mouse (kcal) from NDW (*n* = 6) and SucrW_2d_ (*n* = 6) mice. **C** Baseline total (chow + water) intake per mouse (kcal) from NDW and SucrW_2d_ mice. **D** HFD intake per mouse (kcal) compared to NCD/NDW (*n* = 10) baseline intake following 2d of HFD feeding in SucrW_2d_ (*n* = 8), SucrW_12Wk_ (*n* = 4), and NDW/HFD_2d_ (*n* = 7). **E**. Bodyweight gain during the first two days of SucrW or HFD feeding in NDW (*n* = 9), SucrW_2d_ (*n* = 10), SucrW_2d+HFD_ (*n* = 12), and SucrW_12Wk+HFD_ (*n* = 11). **F**. Plasma leptin during the two days of SucrW or HFD feeding in NDW (*n* = 9), SucrW_2d_ (*n* = 5), SucrW_2d+HFD_ (*n* = 7), and SucrW_12Wk+HFD_ (*n* = 4). **G**. AgRP neuronal firing rate in NDW (*n* = 17), SucrW_2d_ (*n* = 24), SucrW_2d+HFD_ (*n* = 16), and SucrW_12Wk+HFD_ (*n* = 13) following HFD_2d_ feeding. **H** Resting membrane potential of AgRP neurons from SucrW_2d_ and SucrW_12Wk_ with HFD_2d_. **I** Representative traces of NDW, SucrW_2d_, SucrW_2d+2d HFD_, SucrW_12Wk+2d HFD_ mice. **J** Representative trace and change in firing rate (**K**) from bath application of leptin in SucrW_2d+2dHFD_ mice (*n* = 8). **L** Representative trace and change in firing rate (**M**) from bath application of leptin in SucrW_12Wk+2dHFD_ mice (*n* = 8). For all violin plots, dashed line indicates median, dotted lines indicate quartiles. Statistical comparisons using *t*-tests or ordinary one-way ANOVA with a *post hoc* Tukey’s multiple comparisons test. (*p < 0.05; **p < 0.01; ***p < 0.001, ****p < 0.0001).

Previous studies have identified acute HFD feeding as drivers of altered function of arcuate AgRP neurons [28]. To explore this effect in SucrW mice, we provided access to sucrose water for only 2 days (Fig. 6A), which we previously demonstrated to be sufficient to induce hyperexcitability in mice fed a HFD [28]. Similar to SucrW_12Wk_ mice, acute Sucrose fed mice (SucrW_2d_) consumed fewer kcal from chow (Fig. 6B) but more total kcal (chow + water) compared to NDW mice (Fig. 6C). Unlike what we previously observed in 2d HFD feeding, acute SucrW consumption did not significantly increase intrinsic AgRP neuronal activity (*p* = 0.3587; Fig. 6G, H).

To determine whether there is an additive effect of liquid sucrose consumption and HFD, mice given either short-term (2d) or long-term (12wk) access to sucrose water were also given access to HFD for 2 days in place of normal chow (Fig. 6A). Following either short- (SucrW_2d+2d HFD_; *p* < 0.0001) or long-term (SucrW_12Wk+2d HFD_; *p* < 0.0001) sucrose consumption, HFD intake was increased similar to HFD_2d_ feeding alone (HFD_2d_; *p* = 0.0016) compared to NDW mice (Fig. 6D). This corresponded with a trend for increased bodyweight in SucrW_2d+2d HFD_ (*p* = 0.0504) and an increase in bodyweight in SucrW_12Wk+2d HFD_ mice (*p* = 0.0074) not seen in SucrW_2d_ mice (*p* = 0.9179) compared to NDW_fed_ mice and both SucrW_2d_ (*p* = 0.0066) and SucrW_12Wk_ (*p* = 0.0008) weighed more after 2 days of HFD feeding than SucrW_2d_ mice (Fig. 6E). Plasma leptin levels were also increased in SucrW_12Wk+2d HFD_ (*p* = 0.0007) compared to NDW_fed_ mice (Fig. 6F). Acute HFD feeding did not lead to significant differences in plasma insulin or liver weights between NDW_fed_, SucrW_2d_, SucrW_2d+2d HFD_, and SucrW_12Wk+2d HFD_ mice (Fig. S5A, B). Unlike acute sucrose alone, acute HFD feeding following Sucrose consumption (SucrW_2d+2d HFD_, *p* = 0.0155; SucrW_12Wk+2d HFD_, *p* = 0.0307) did significantly increase AgRP neuronal activity compared to NDW_fed_ mice (Fig. 6G–I), with no difference in RMP (Fig. 6H, I). The increased baseline firing in SucrW_2d+2d HFD_ mice did not correspond to a deficiency in leptin signaling (Fig. 6J, K) like acute HFD feeding alone [28]. However, SucrW_12Wk+2d HFD_ mice had an attenuated leptin response (Fig. 6L, M), similar to SucrW_12Wk_ and DIO mice [24].

## Discussion

As the obesity crisis remains one of the largest public health concerns in developed countries, safe, effective, and lasting therapeutics remain elusive [5]. While considerable research has highlighted the importance of neuronal regulation of food intake and obesity [3, 6], the influence of dietary components on the CNS has received less attention. Most research has focused on the effects of high-fat diets [24, 29–31] on driving DIO and we have identified that diet composition (not necessarily caloric intake) may promote DIO [28]. Here, we show that high sugar diet drives increased caloric intake and AgRP neuronal activity, though not to the extent of HFD [24]. When given HFD for 2 days, caloric intake, bodyweight gain, and AgRP neuronal activity match that seen in HFD fed mice, suggesting that more AgRP neurons respond to fat + sugar than to sugar alone. While other components typical of the western diet (carbohydrates, fats, and proteins) [69] remain to be investigated, we identify a link between sugar consumption and AgRP neuronal activity in the absence of weight gain and hyperphagic behavior.

Consistent with previous studies of sugar sweetened water diets [37–39], we did not identify significant changes in bodyweight or body composition in SucrW_12Wk_ mice. This is likely due to (1) decreases in NCD intake as mice attempt to regulate total caloric intake in response to increased SucrW intake, (2) robust sucrose preference, (3) gastric distention from liquid intake that prevents significant solid diet intake, or a combination of these factors [37–40, 42]. While fat mass was unchanged in SucrW mice, lean mass was decreased; this decoupling between fat and lean mass has been described previously in studies examining the function of AgRP neurons and the ghrelin receptor (GHSR1) [70–74]. Blood glucose is slightly elevated in SucrW_12Wk_ mice while plasma leptin and insulin levels remain comparable to lean mice. Caloric intake following a fast further suggests that peripheral maintenance of bodyweight and food intake remain intact [75, 76]. However, liver weight was elevated in SucrW_12Wk_ mice, suggesting an increase in hepatic fat content and impaired liver function [37], though this weight difference was reversed following a fast. Overall, these differences suggest that CNS control of food and caloric intake may be altered in SucrW_12Wk_ mice. Based on these differences in caloric preference, we investigated the role of putative nutrient sensing neurons in the ARH of the hypothalamus.

AgRP neurons play a key role in the integration of peripheral and central signals and their activity is tied to food intake and body weight [19–23, 25]. Previous studies from our lab and others have identified DIO-related changes in AgRP neuronal activity and function linked to synaptic and intrinsic remodeling. Wei et al., 2015, provide evidence that diet composition may be sufficient to alter baseline AgRP neuronal activity that precedes any changes in bodyweight or peripheral hormone disruption. In accordance, we found that SucrW_12Wk_ feeding increased AgRP neuronal activity and caloric intake without significantly altering bodyweight or body composition. These changes in neuronal firing mirror HFD-induced changes to AgRP neuronal firing rate in DIO mice [24, 31], though to a lesser extent, likely driven by functional differences in fat- and sugar-sensitive afferents to the hypothalamus and AgRP neurons [33–35, 77] or even disparate circuitries for hedonic and nutritional sugar preference [40]. Interestingly, fasting failed to further increase the F_AP_ of AgRP neurons suggesting that (1) AgRP neurons are refractory to additional relevant stimuli, or (2) this rate represents a ceiling beyond which AgRP neurons are not able to sustain action potential firing. Our analysis of intrinsic AgRP neuronal firing distribution suggests that diet manipulation (SucrW, fast, HFD) does not change the maximum F_AP_ of AgRP neurons, instead shifting the lower and medium firing populations to a higher ‘set-point’ [24, 76].

Additionally, leptin signaling was functionally altered in SucrW_12Wk_ fed mice, an effect previously described in HFD fed mice utilizing both ex-vivo electrophysiological recordings [24] and *p*-STAT3 activation [78] and in rats fed sugar sweetened water [38]. Future research should explore the potential mechanisms of diet induced leptin resistance in the absence of obesity (but in the presence of AgRP neuronal hyperexcitability). Our results suggest that rather than inducing complete insensitivity to the inhibitory effects of leptin, long-term consumption of SucrW attenuates the response of AgRP neurons to leptin. We found that most AgRP neurons from SucrW mice exhibited some degree of inhibition; however, compared to the NDW controls, this response was more variable, with some AgRP neurons exhibiting a high level of activity after administration of leptin, suggesting an incomplete or attenuated leptin sensitivity. As phosphorylation of STAT3 in response to leptin has also been shown to be altered in HFD-induced obese animals, evaluation of *p*-STAT3 activation may offer additional insight into the broader response of these signaling pathways to leptin in SucrW treated mice. Further, synaptic plasticity of excitatory and inhibitory inputs to AgRP neurons in lean SucrW_12Wk_ mice were akin to DIO HFD_8Wk_ fed mice [31]. Briefly, decreased mEPSC and mIPSC amplitudes in SucrW_12Wk_ mice suggest that AgRP response to synaptic input is altered compared to NDW_fed_ mice. Increased mIPSC frequency in lean SucrW_12Wk_ mice reflects previously described plasticity of these inputs [66, 67]. Specifically, inhibitory inputs from the ventral compartment of the dorsomedial nucleus of the hypothalamus (vDMH) [25] and the anterior bed nuclei of the stria terminalis (aBNST) [79] to AgRP neurons have been identified. While AgRP afferent neurons in the vDMH appear to be diet sensitive [80–82], the response of presynaptic aBNST neurons to diet manipulation remains unclear. These data support previous evidence that Sucrose consumption alters synaptic connectivity [36] and the ‘top-down’ mechanisms to inhibit persistently activated AgRP neurons function without obesity and are similar to DIO mice both ex vivo [31] and in vitro [29, 30].

Predictably, SucrW_2d_ mice preferred Sucrose water to NCD and consumed more calories than NDW mice. Similar to previous studies [83–85], we show that short- and long-term Sucrose feeding increased HFD intake, slightly exceeding the hyperphagic behavior seen during the first 2 days of HFD feeding. This corresponded with trending and significant increases in bodyweight of SucrW_2d+2d HFD_ and SucrW_12Wk+2d_ HFD mice, respectively which precedes bodyweight changes from acute Sucrose or HFD [28]. AgRP neuronal activity was increased in both SucrW_2d+2d HFD_ and SucrW_12Wk+2d HFD_ groups, though this effect was predicted as both SucrW_12Wk_ and HFD_2d_ are sufficient to increase AgRP neuronal activity alone [28]. Finally, we found that leptin signaling was intact in lean SucrW_2d+2d HFD_, which aligns with our prior slice electrophysiology data [28] but not biochemical assays of leptin signaling [86, 87]. SucrW_12Wk+2d HFD_ mice had elevated plasma leptin and an attenuated leptin response, while SucrW_12Wk_ mice that had not consumed HFD had normal plasma leptin and an attenuated leptin response.

## Limitations

The current study was limited to observing the effects of Sucrose consumption on ex vivo AgRP neurons. Future studies will consider the impact of diet and specific macronutrient sensing in the gut using in vivo calcium imaging techniques following both short- and long-term feeding schedules as has been previously established in studies using HFD [29, 30]. Further, these experiments should be conducted longitudinally with more precise automated systems to measure diet and water consumption [88], allowing for quantification of individual caloric intake. While we utilized a group housing model to reduce isolation stress [57–59], future studies should incorporate individual housing for more precise quantification of individual intake. Further, it is understood that Sucrose consumption can alter the magnitude of astrocyte and microglia inflammation [89] and this may influence intrinsic and synaptic plasticity of AgRP neurons [90]. While C57Bl/6 J female mice are more resistant to DIO than males [31, 91] and did not display metabolic changes on SucrW_12Wk_ diet, future studies should explore sex-specific resistance factors in females that might be associated with functional or synaptic changes in AgRP neuron populations. Finally, the influence of hypothalamic function on throughout the lifespan should be considered; as consumption of diets high in Sucrose or other obesogenic macronutrients likely have profound effects on early- [92–94] and later-life [95–99] hypothalamic and AgRP neuronal function.

## Conclusions

We have identified effects of short- and long-term Sucrose consumption on bodyweight and food intake, along with intrinsic and synaptic plasticity changes of AgRP neurons. Combined, these data suggest changes in AgRP neuronal function coincide with increased calorie intake and precede differences in bodyweight. Long-term sucrose consumption does not alter AgRP neuronal function to the same extent as HFD, suggesting differential innervation of specific gut-brain afferents. Further, Sucrose feeding for 2-days or 12-weeks augmented HFD intake and bodyweight gain. This effect corresponded with increased AgRP neuronal activity. Overall, this highlights the important role for top-down regulation of neuronal circuits that regulate food intake and bodyweight, supporting hypotheses that therapeutics for obesity will require ‘resetting’ of homeostatic circuitries within the CNS.

## Supplementary information

Supplemental Figure Legends

Supplemental Figure S1

Supplemental Figure S2

Supplemental Figure S3

Supplemental Figure S4

Supplemental Figure S5

## Data Availability

All relevant data and analysis tools are available upon reasonable request from the authors.
