# Supplementary figures and images for: High sucrose consumption decouples intrinsic and synaptic excitability of AgRP neurons without altering body weight
Source: Int J Obes (Lond). 2023 Feb 1;47(3):224–35. doi: 10.1038/s41366-023-01265-w (PMC10023568; doi:10.1038/s41366-023-01265-w)

# Figure S1

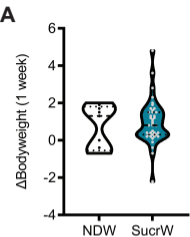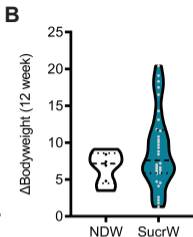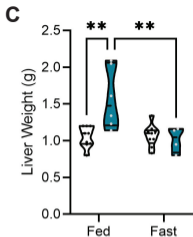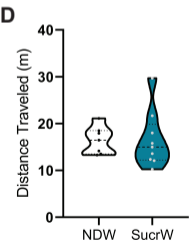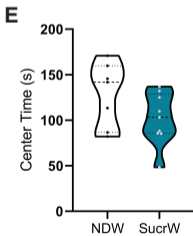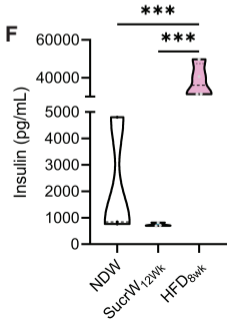

Supplement: Supplementary file 1 — Supplemental Figure Legends [file 41366_2023_1265_MOESM1_ESM.pdf]

# Figure S2

A

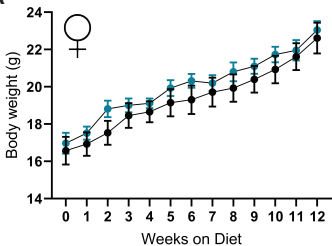

B

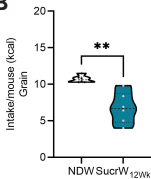

D

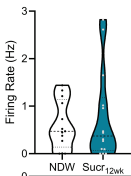

C

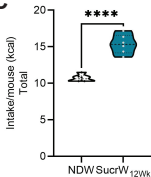

E

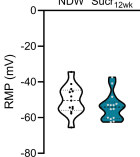

Supplement: Supplementary file 2 — Supplemental Figure S1 [file 41366_2023_1265_MOESM2_ESM.pdf]

# Figure S4

**A**

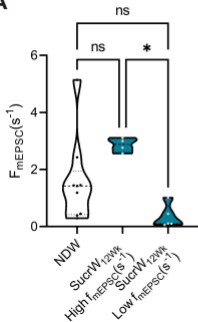

**B**

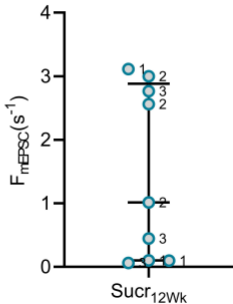

Supplement: Supplementary file 4 — Supplemental Figure S3 [file 41366_2023_1265_MOESM4_ESM.pdf]

# Figure S5

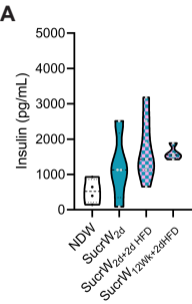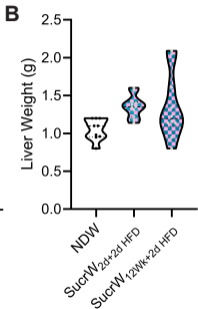

Supplement: Supplementary file 5 — Supplemental Figure S4 [file 41366_2023_1265_MOESM5_ESM.pdf]
