# Supplementary material for: High sucrose consumption decouples intrinsic and synaptic excitability of AgRP neurons without altering body weight
Source: Int J Obes (Lond). 2023 Feb 1;47(3):224–35. doi: 10.1038/s41366-023-01265-w (PMC10023568; doi:10.1038/s41366-023-01265-w)
Supplement: Supplementary file 3 — Supplemental Figure S2 [file 41366_2023_1265_MOESM3_ESM.pdf]

Figure S3

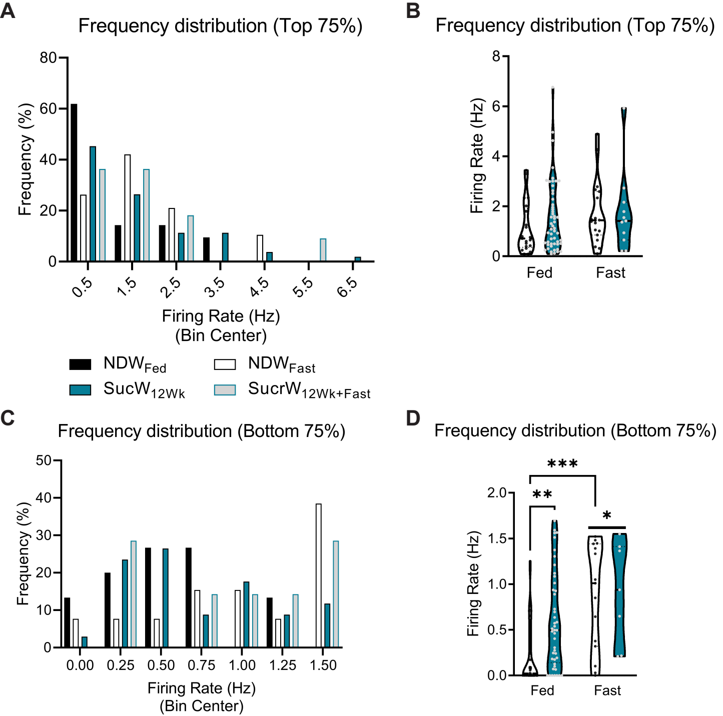
A
Frequency distribution (Top 75%)
Frequency (%)
80
60
40
20
0
0.5
1.5
2.5
3.5
4.5
5.5
6.5
Firing Rate (Hz)
(Bin Center)
B
Frequency distribution (Top 75%)
Firing Rate (Hz)
8
6
4
2
0
Fed
Fast
NDW Fed
NDW Fast
SucW 12Wk
SucrW 12Wk+Fast
C
Frequency distribution (Bottom 75%)
Frequency (%)
50
40
30
20
10
0
0.00
0.25
0.50
0.75
1.00
1.25
1.50
Firing Rate (Hz)
(Bin Center)
D
Frequency distribution (Bottom 75%)
***
**
*
Firing Rate (Hz)
2.0
1.5
1.0
0.5
0.0
Fed
Fast
