# Supplementary material for: High sucrose consumption decouples intrinsic and synaptic excitability of AgRP neurons without altering body weight
Source: Int J Obes (Lond). 2023 Feb 1;47(3):224–35. doi: 10.1038/s41366-023-01265-w (PMC10023568; doi:10.1038/s41366-023-01265-w)
Supplement: Supplementary file 6 — Supplemental Figure S5 [file 41366_2023_1265_MOESM6_ESM.docx]

**Figure S1**: **A**. There was no difference in bodyweight after one or twelve weeks (**B**) of SucrW (n=39, 33 respectively) consumption compared to NDW mice (n=9, 6 respectively). **C.** Wet liver weight (g) was elevated in fed SucrW mice (n=6) compared to fed NDW (fed: n=9, fast: 9) and fasted SucrW (fast: 4) mice. **D.** Plasma insulin is elevated in HFD_8wk_ (n=4) compared to NDW (n=3) and SucrW_12Wk_ mice (n=3). No difference in distance travelled (**F**) or time spent in the center (**G**) of the OFT between NDW (n=7) and SucrW_12Wk_ mice (n=9). Statistical comparisons using t-tests or ordinary one-way ANOVA with a *post hoc* Tukey’s multiple comparisons test. (** = p < 0.01; *** = p < 0.001).

**Figure S2: A**. Female mice do not gain weight on SucrW12Wk (n=10) compared to NDW female mice (n=11). **B.** Baseline chow (NDW: n=6, SucrW: 6) intake was decreased in SucrW fed female mice but total caloric intake (NDW: n=6 SucrW: 6) (kcal) was increased in SucrW fed female mcie (**C**). **D.** There was no difference in AgRP neuronal firing rate or RMP (**E**) in SucrW fed female mice, compared to NDW fed control females. For all violin plots, dashed line indicates median, dotted lines indicate quartiles. For all analysis, diet conditions compared to NDW using standard t-test or parametric ANOVA with a *post hoc* Tukey’s multiple comparisons test and a mixed-effects RM ANOVA for repeated measures (** = p < 0.01; **** = p < 0.0001).

**Figure S3: A**. Quartiles were determined for the entire pooled group of neurons (F_AP_ < 25 percentile; 0.08659 Hz), medium firing (F_AP_ > 25 and < 75 percentile), and high firing (F_AP_ > 75 percentile; 1.75 Hz). Frequency distribution of AP firing frequency in AgRP neurons from male NDW and SucrW mice with including only the top 75% F_AP_ neurons and subsequent loss of main effect of diet and fasting (**B**). **C.** Frequency distribution of AP firing frequency in AgRP neurons from male NDW and SucrW mice with including only the bottom 75% F_AP_ neurons and subsequent main effect of diet and fasting (**D**). For all violin plots, dashed line indicates median, dotted lines indicate quartiles. For all analysis, diet conditions compared to NDW using parametric ANOVA with a *post hoc* Tukey’s multiple comparisons test (* = p < 0.05; ** = p < 0.01; **** = p < 0.0001).

**Figure S4: A.** A median split of mEPSC data from SucrW_12Wk_ mice (Figure 4B) reveals significantly different low (n=5) and high (n=4) frequency inputs that are not different from NDW (n=9), with representation from each mouse in both the low and high F_mEPSC_ groups (**B**). For violin plots, dashed line indicates median, dotted lines indicate quartiles. For all analysis, diet conditions compared to NDW using parametric ANOVA with a *post hoc* Tukey’s multiple comparisons (* = p < 0.05).

**Figure S5: A.** Plasma insulin does not change significantly in SucrW_2d_ (n=4), SucrW_2d+HFD_ (n=7), or SucrW_12Wk+HFD_ (n=4) following HFD_2d_ feeding compared to NDW (n=4) mice. (B**.**) Liver weight does not change significantly in SucrW_2d+HFD_ (n=11), and SucrW_12Wk+HFD_ (n=10) following HFD_2d_ feeding compared to NDW (n=9) mice. For all violin plots, dashed line indicates median, dotted lines indicate quartiles. For all analysis, diet conditions compared to NDW using parametric ANOVA with a *post hoc* Tukey’s multiple comparisons test.
